# Postoperative diastolic perfusion pressure as a predictor of delirium after cardiac surgery

**DOI:** 10.3389/fcvm.2026.1736972

**Published:** 2026-05-08

**Authors:** Xiao Shen, Zifan Wang, Liang Hong, Jiakui Sun, Wenxiu Chen, Xiang Wang, Cui Zhang

**Affiliations:** 1 Department of Critical Care Medicine, Nanjing First Hospital, Nanjing Medical University, Nanjing, China; 2 Nanjing Medical University, Nanjing, China

**Keywords:** cardiac surgery, diastolic perfusion pressure, hemodynamic monitoring, postoperative delirium, predictive model

## Abstract

**Background:**

Postoperative delirium (POD) is a frequent neurological complication after cardiac surgery, associated with increased morbidity, prolonged hospitalization, and poor long-term outcomes. Diastolic perfusion pressure (DPP), reflecting the difference between diastolic arterial pressure and central venous pressure (CVP), plays a critical role in organ perfusion, yet its association with POD remains unclear. This study aimed to investigate the predictive value of postoperative DPP for POD in patients undergoing cardiac surgery with cardiopulmonary bypass.

**Methods:**

In this retrospective case-control study, 4,418 adult patients who underwent cardiac surgery between May 2019 and December 2022 were analyzed. POD was assessed using the Confusion Assessment Method for the Intensive Care Unit (CAM-ICU). Postoperative DPP was calculated as diastolic arterial pressure minus CVP, and the mean values during the first 6 and 12 postoperative hours (DPP_6h_mean, DPP_12h_mean) were extracted. Multivariable logistic regression, restricted cubic spline (RCS) analysis, and a predictive nomogram were used to evaluate the association between DPP and POD.

**Results:**

POD occurred in 352 patients (8.0%). Compared with those without POD, patients with delirium had significantly lower postoperative DPP values at both 6 and 12 h after surgery (all *P* < 0.001). In multivariable analysis, male sex, older age, higher APACHE II score, longer operation time, and lower DPP_12h_mean were independent predictors of POD. Each 5 mmHg decrease in DPP_12h_mean increased the risk of delirium by approximately 20% (adjusted OR = 1.20, 95% CI: 1.06–1.35, *P* = 0.004). The predictive model incorporating DPP_12h_mean achieved good discrimination (AUROC = 0.743) and clinical utility.

**Conclusions:**

Lower postoperative diastolic perfusion pressure is independently associated with an increased risk of postoperative delirium after cardiac surgery. DPP may serve as a clinically useful marker reflecting effective organ perfusion. Optimization of DPP may represent a potential strategy for reducing delirium risk; however, this requires confirmation in prospective studies.

## Introduction

Postoperative delirium (POD) is a common but multifactorial neurological complication after cardiac surgery (1), with reported incidences ranging from 10% to 40%, depending on patient characteristics and diagnostic criteria (2, 3). Its pathogenesis is complex, involving multiple interacting mechanisms such as cerebral hypoperfusion, systemic inflammation, and hemodynamic instability (4, 5). With the aging population and rapid expansion of cardiac surgical procedures—particularly among individuals aged ≥65 years in China—the burden of POD has become an increasingly important clinical concern.

POD is associated with a range of adverse outcomes (6, 7). In the short term, it leads to prolonged intensive care unit (ICU) and hospital stays, increased healthcare costs, and greater resource utilization. Long-term consequences include persistent cognitive decline, higher risk of dementia, functional impairment, and increased mortality. Given these substantial impacts, early identification of modifiable risk factors and implementation of targeted preventive strategies are crucial.

A growing body of evidence has identified multiple perioperative risk factors for POD, including advanced age, baseline cognitive impairment, intraoperative cerebral hypoperfusion, systemic inflammation, and perioperative hemodynamic instability. In particular, studies focusing on neurocognitive function and brain injury have demonstrated that disturbances in cerebral perfusion and oxygenation are closely associated with the development of delirium after cardiac surgery. In addition, inflammatory responses and blood–brain barrier dysfunction have also been implicated as key contributors to POD (4, 5).

Among the various contributors to POD, hemodynamic instability has been increasingly recognized as a key modifiable determinant. Recent studies have shown that intraoperative and postoperative hypotension, as well as excessive blood-pressure variability, are associated with a higher incidence of POD following cardiac surgery (8–10). Furthermore, several studies have explored the relationship between systemic hemodynamic parameters and neurological outcomes, suggesting that both inadequate arterial pressure and impaired perfusion may contribute to delirium risk. However, most of these studies have focused primarily on mean arterial pressure (MAP), which may not adequately represent the true perfusion pressure at the organ level.

Diastolic perfusion pressure (DPP)—defined as the difference between diastolic arterial pressure (DAP) and central venous pressure (CVP)—reflects the effective pressure gradient driving blood flow during diastole. It is particularly relevant for low-resistance, high-diastolic-flow organs such as the brain and kidneys. Previous investigations have demonstrated that reduced diastolic pressure or DPP is independently associated with organ dysfunction and mortality among critically ill patients (11, 12), sometimes exerting a stronger predictive value than MAP alone. Moreover, a lower postoperative nadir DPP has been identified as an independent risk factor for acute kidney injury (AKI) after cardiac surgery (13).

In terms of neurological outcomes, low diastolic pressure has been linked to delirium in patients with circulatory shock, even after adjustment for MAP (14), underscoring the critical role of diastolic perfusion in maintaining adequate cerebral blood flow under hemodynamic stress. Similarly, higher systemic perfusion pressures during cardiopulmonary bypass (CPB)—which better preserve diastolic flow—have been associated with a reduced incidence of postoperative delirium and cognitive dysfunction (15).

Despite these advances, current evidence has largely focused on general hemodynamic parameters or intraoperative conditions, and studies specifically evaluating the role of early postoperative diastolic perfusion pressure in relation to delirium after cardiac surgery remain limited. Given that the early postoperative period is characterized by dynamic hemodynamic changes and increased vulnerability of cerebral perfusion, this gap warrants further investigation.

Collectively, these findings suggest that maintaining adequate diastolic perfusion pressure may be crucial for preventing both organ dysfunction and POD in cardiac surgical patients. However, evidence specifically addressing the association between postoperative DPP and delirium after cardiac surgery remains limited. Therefore, the present study aimed to evaluate the relationship between postoperative DPP and the occurrence of delirium in patients undergoing cardiac surgery.

## Materials and methods

### Study design and setting

This retrospective case-control study was conducted in the Cardiovascular Intensive Care Unit (CVICU) of Nanjing First Hospital, a tertiary teaching hospital affiliated with Nanjing Medical University. The study was conducted in accordance with the principles of the Declaration of Helsinki and approved by the Ethics Committee of Nanjing First Hospital, Nanjing Medical University (KY20220518-01-KS-01). The requirement for informed consent was waived owing to the retrospective nature of the study.

### Patient selection

Adult patients (≥18 years) who underwent cardiac surgery with CPB and were admitted to the CVICU between May 2019 and December 2022 were screened. Exclusion criteria were: postoperative delirium occurred >7 days after cardiac surgery, preoperative ICU admission, died during or within 48 h after surgery, ICU stay <12 h after cardiac surgery, unable to communicate due to pre-existing neurological disorders including dementia, stroke or other cerebral diseases, or incomplete hemodynamic or CAM-ICU data.

### Anesthesia management

All patients underwent cardiac surgery under general anesthesia administered by a dedicated and experienced cardiac anesthesia team using a standardized institutional protocol. Anesthesia was typically induced with intravenous agents (e.g., midazolam, etomidate, or propofol) in combination with opioids, and maintained with a combination of intravenous and/or inhalational anesthetics, along with neuromuscular blocking agents as required.

Intraoperative hemodynamic management aimed to maintain adequate organ perfusion according to standard clinical practice, including the use of vasopressors and inotropic support when necessary. Anesthetic depth was routinely managed according to institutional standards and clinical judgment, although specific monitoring parameters (e.g., bispectral index) were not consistently recorded in the database.

Postoperative sedation and analgesia were conducted according to standardized ICU protocols to ensure patient comfort and hemodynamic stability.

Overall, the anesthesia and sedation strategies were relatively consistent across patients, which may have minimized variability in anesthetic exposure and reduced potential confounding effects.

### Data collection

Demographics, comorbidities, APACHE II and euroSCORE, intraoperative variables (surgery type, duration, CPB and aortic cross-clamp times, fluid balance) and continuous invasive blood pressure were extracted from the Electronic Medical Records (EMR) database and analyzed. Postoperatively, hemodynamics—including systolic, diastolic, and mean arterial pressure, heart rate (HR), CVP, and peripheral capillary oxygen saturation (SpO₂)—were extracted for the first 12 h. Additional data included laboratory results, maximum inotropic support (norepinephrine, dopamine, epinephrine, etc.), and use of mechanical circulatory devices (intra-aortic balloon pump[IABP], extracorporeal membrane oxygenation [ECMO]). Clinical outcomes included mechanical ventilation (MV) duration, ICU and hospital length of stay, hospital mortality, incidence of AKI and postoperative atrial fibrillation (POAF), and need for renal replacement therapy (RRT).

### Hemodynamic monitoring and DPP calculation

Intraoperative blood pressure and CVP were continuously monitored via invasive arterial and central venous catheters, with recordings every five minutes using the anesthesia monitoring system (DoCare). Postoperatively, invasive systolic (SAP), diastolic (DAP), and mean arterial pressure (MAP) and CVP were continuously measured during the first 12 h in the ICU and documented every 30 min in EMR database. Extreme values—systolic pressure <40 mmHg or >300 mmHg, and diastolic pressure <20 mmHg or >150 mmHg—were considered physiologically implausible and excluded from analysis. Extreme CVP values (<0 mmHg or >30 mmHg) were considered invalid and excluded. Only valid paired measurements of arterial pressure and CVP were used for further analysis.

DPP was defined as the difference between DAP and CVP, as previous reported (16):

DPP=DAP−CVP

For analysis, the mean DPP was calculated over the first 6 and 12 h postoperatively to reflect overall organ perfusion. Only valid paired measurements of DAP and CVP were included, with missing or physiologically implausible values excluded. These metrics, designated as DPP_6h_mean and DPP_12h_mean, were subsequently analyzed for their association with POD.

### Outcome definition and delirium assessment

The primary outcome was POD, defined as delirium occurring within 7 days after cardiac surgery. POD was systematically evaluated twice daily during the ICU stay and on an as-needed basis after ICU discharge using the Confusion Assessment Method for the ICU (CAM-ICU) (17). Patients' level of consciousness was first assessed with the Richmond Agitation-Sedation Scale (RASS), and CAM-ICU assessments were performed in patients with RASS ≥ −3. Delirium was diagnosed if acute or fluctuating mental status changes and inattention were present, along with either disorganized thinking or altered consciousness. Secondary outcomes included ICU and hospital length of stay, incidence of AKI, requirement for renal replacement therapy (RRT), and in-hospital mortality. All assessments were conducted by trained nurses, and findings were documented in the electronic medical record to allow continuous monitoring of delirium incidence and duration throughout the postoperative period.

### Statistical analyses

All statistical analyses were performed using R software (version 4.3.3, R Foundation for Statistical Computing, Vienna, Austria). Continuous variables were expressed as mean ± standard deviation (SD) or median with interquartile range (IQR), as appropriate, and categorical variables were presented as counts and percentages. Group comparisons were conducted using Student's *t*-test or Wilcoxon rank-sum test for continuous variables, and chi-square or Fisher's exact test for categorical variables. Violin plots were generated to visualize the distribution of DPP_6h_mean and DPP_12h_mean between the two groups.

Univariate logistic regression was initially performed to identify potential predictors of POD, including demographic and perioperative variables. All variables considered clinically relevant or potentially associated with POD were subsequently entered into the multivariable logistic regression model, regardless of their statistical significance in univariate analysis. To address potential multicollinearity, among correlated variables (e.g., DPP vs. CVP, DAP, or MAP), only DPP was retained in the final model. This full-model approach was used to adjust for potential confounders and to estimate the independent effect of each variable. Multicollinearity was further assessed using variance inflation factors (VIF), with no significant collinearity detected among the included variables. A clinical prediction model was then developed based on independent predictors identified in the multivariable analysis. Model performance was evaluated in terms of discrimination, calibration, and clinical utility using the area under the receiver operating characteristic curve (AUROC), calibration curves, and decision curve analysis (DCA), respectively. Restricted cubic spline (RCS) analysis was further applied to explore potential nonlinear associations between DPP and POD. A nomogram was constructed to provide individualized risk estimation for POD.

Subgroup analyses were performed to assess the consistency of the association across clinically relevant strata, including age, sex, APACHE II score, and operation time. In addition, the effect of DPP_12h_mean on POD risk was quantified per 5 mmHg decrease, both before and after adjustment for age, sex, APACHE II score, and operation time. A two-sided *P* value <0.05 was considered statistically significant.

## Results

### Study population

**Figure 1:**
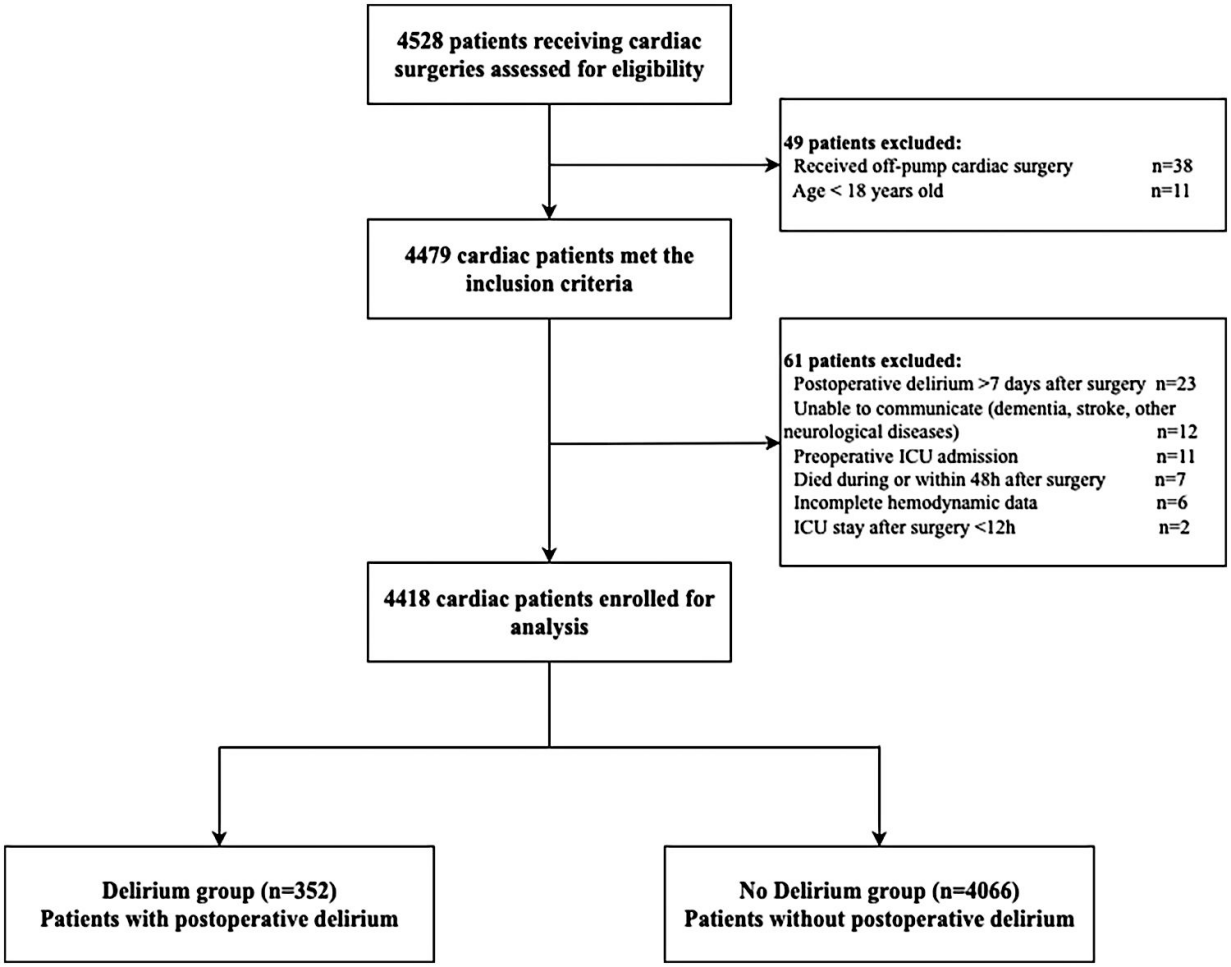
Flowchart of patient selection for the study. The diagram illustrates the inclusion and exclusion process used to identify the final study cohort for postoperative delirium analysis.

A total of 4,528 patients who underwent cardiac surgery were screened between May 2019 and December 2022 (Figure 1). After applying the predefined inclusion and exclusion criteria, 4,418 patients were eligible for the final analysis, including 352 patients who developed POD (Delirium group) and 4,066 patients without POD (No Delirium group). The overall incidence of POD was 8.0% (352/4,418), most commonly occurring on postoperative day 3 (range, 1–7 days).

### Baseline characteristics

Baseline characteristics of the study population are summarized in Table 1. Patients who developed POD were typically older and more frequently male compared with those without POD. They exhibited higher Acute Physiology and Chronic Health Evaluation II (APACHE II) and European System for Cardiac Operative Risk Evaluation (EuroSCORE) scores, as well as a greater burden of comorbidities, with the exception of atrial fibrillation (AF) and chronic obstructive pulmonary disease (COPD).

**Table 1:** Baseline characteristics of the patients with or without postoperative delirium after cardiac surgery.

Variable	No Delirium (*n* = 4,066)	Delirium (*n* = 352)	*P* value
Age, y	63 [54–70]	68 [61–74]	<0.001
BMI, kg/m^2^	20.20 [19.03–21.48]	19.72 [18.81–22.03]	0.516
Male, *n* (%)	2,412 (59.3%)	256 (72.7%)	<0.001
APACHE II score	12 [10–15]	14 [12–16]	<0.001
EuroSCORE	5 [4–6]	6 [5–7]	<0.001
Co-morbidities, *n* (%)			
Stroke	474 (11.7%)	57 (16.2%)	0.015
Hypertension	1,955 (48.1%)	195 (55.4%)	0.010
Diabetes mellitus	902 (22.2%)	96 (27.3%)	0.034
CHD	1,737 (42.7%)	208 (59.1%)	<0.001
CRF	158 (3.9%)	43 (12.2%)	<0.001
AF	895 (22.0%)	75 (21.3%)	0.811
COPD	179 (4.4%)	15 (4.3%)	1.000
Surgery types, *n* (%)			
Valve surgery	1,737 (42.7%)	104 (29.5%)	<0.001
CABG	1,174 (28.9%)	127 (36.1%)	0.005
Combined valve surgery and CABG	367 (9.0%)	56 (15.9%)	<0.001
Aortic surgery	418 (10.3%)	32 (9.1%)	0.538
Other surgeries	308 (7.6%)	9 (2.6%)	0.001
Aortic arch replacement	60 (1.5%)	24 (6.8%)	<0.001
Intra-operative indexes			
Operation time, min	250.0 [215.0–295.0]	285.0 [240.0–330.0]	<0.001
CPB time, min	109.0 [84–138]	125.0 [98.7–160.5]	<0.001
Aortic cross-clamp time, min	75.0 [57.0–100.0]	82.5 [65.0–113.0]	<0.001
Total fluid input, ml	2,000 [1,500–2,000]	2,000 [1,500–2,500]	<0.001
Blood transfusion,ml	1,150 [900–1,500]	1,300 [1,000–1,728]	<0.001
Total fluid output, ml	1,950 [1,600–2,400]	2,100 [1,638–2,555]	0.008
Blood loss, ml	1,000 [1,000–1,300]	1,200 [1,000–1,400]	<0.001
Urine output, ml	800 [550–1,200]	800 [500–1,200]	0.681
Fluid balance, ml	−800 [−1,650–−100]	−700 [−1,550–−48]	0.164
Laboratory results on ICU admission			
WBC, *109/L	11.00 [8.63–13.82]	10.76 [8.55–13.92]	0.699
HCT, %	28.00 [25.00–31.50]	27.20 [24.10–30.05]	<0.001
PLT, *109/L	121.00 [97.00–151.00]	119.00 [94.00–156.00]	0.751
Hb, g/L	94.00 [83.00–105.00]	90.00 [80.00–101.00]	<0.001
CRP, μg/L	77.68 [38.76–117.56]	79.70 [46.47–119.90]	0.443
PaO_2_, mmHg	126.20 [99.90–163.80]	118.10 [95.05–156.45]	0.007
Lac, mmol/L	1.50 [1.00–2.40]	1.80 [1.10–3.15]	<0.001
BUN, mmol/L	6.90 [5.69–8.44]	7.44 [6.05–9.54]	<0.001
Creatinine, μmol/L	73.40 [61.10–88.20]	82.56 [67.25–103.12]	<0.001
PCT, ng/ml	0.10 [0.04–0.41]	0.37 [0.08–1.68]	<0.001
CK, U/L	276.00 [194.50–395.00]	290.50 [195.00–425.75]	0.155
CK-MB, U/L	33.00 [22.00–54.00]	33.00 [22.00–60.00]	0.269
LDH, U/L	542.00 [334.00–741.00]	611.00 [403.00–812.00]	<0.001
TBIL, μmol/L	15.00 [10.60–21.70]	14.30 [10.40–22.40]	0.863
Albumin, g/L	33.10 [30.30–36.10]	32.70 [30.10–36.20]	0.229
Inotropic drugs			
Norepinephrine, μg/kg/min	0.00 [0.00–0.06]	0.05 [0.00–0.15]	<0.001
Dopamine, μg/kg/min	0.00 [0.00–3.00]	0.00 [0.00–3.00]	0.622
Dobutamine, μg/kg/min	0.00 [0.00–3.00]	0.00 [0.00–3.00]	0.006
Epinephrine, μg/kg/min	0.00 [0.00–0.00]	0.00 [0.00–0.03]	<0.001
Milrinone, μg/kg/min	0.00 [0.00–0.00]	0.00 [0.00–0.00]	<0.001
Olprinone, μg/kg/min	0.00 [0.00–0.00]	0.00 [0.00–0.00]	0.107
Levosimendan, μg/kg/min	0.00 [0.00–0.00]	0.00 [0.00–0.00]	<0.001
Hypophysin, U/h	0.00 [0.00–0.00]	0.00 [0.00–0.00]	<0.001
IABP, *n* (%)	53 (1.3%)	26 (7.4%)	<0.001
ECMO, *n* (%)	6 (0.1%)	2 (0.6%)	0.129
Prognosis indexes			
MV duration, h	9.2 [7.0–13.5]	18.1 [10.3–38.3]	<0.001
Length of ICU stay, d	1 [1–2]	3 [2–5]	<0.001
Length of hospital stay, d	17 [14–20]	20 [16–26]	<0.001
In-hospital mortality, *n* (%)	59 (1.5%)	30 (8.5%)	<0.001
Postoperative complications			
AKI, *n* (%)	1,008 (24.8%)	172 (48.9%)	<0.001
RRT requirement, *n* (%)	59 (1.5%)	22 (6.2%)	<0.001
POAF, *n* (%)	2,128 (52.3%)	249 (70.7%)	<0.001

BMI, body mass index; APACHE II, Acute Physiology; Age; Chronic Health Evaluation II; EuroSCORE, European system for cardiac operative risk evaluation; CHD, coronary heart disease; CRF, chronic renal failure; AF, atrial fibrillation; COPD, chronic obstructive pulmonary disease; CABG, Coronary Artery Bypass Grafting; CPB, cardiopulmonary bypass; WBC, white blood cells; HCT, hematocrit; PLT, Platelets; Hb, hemoglobin; CRP, C-Reactive Protein; PaO_2_, partial pressure of oxygen; Lac, lactate; BUN, Blood Urea Nitrogen; PCT, procalcitonin; CK, Creatine Kinase; CK-MB, Creatine Kinase-MB Isoenzyme; LDH, Lactate dehydrogenase; TBIL, total bilirubin; IABP, intra-aortic balloon pump; ECMO, extracorporeal membrane oxygenation; MV, mechanical ventilation; ICU, intensive care unit; AKI, acute kidney injury; RRT, renal replacement therapy; POAF, postoperative atrial fibrillation.

Regarding surgical characteristics, patients with POD were more likely to undergo coronary artery bypass grafting (CABG), combined valve and CABG procedures, and aortic arch replacement. Intraoperatively, they experienced longer operation, CPB, and aortic cross-clamp times, accompanied by increased transfusion requirements.

On admission, laboratory findings indicated lower hematocrit (HCT), hemoglobin (Hb), and arterial oxygen tension (PaO₂), along with elevated levels of lactate, blood urea nitrogen (BUN), creatinine, procalcitonin, and lactate dehydrogenase (LDH). Additionally, these patients required higher inotropic support and more frequent use of IABP assistance.

Clinically, the POD group had significantly longer durations of mechanical ventilation, ICU stay, and total hospitalization, as well as higher rates of postoperative complications and mortality.

### Logistic regression model on hemodynamic indexes and POD

**Figure 2:**
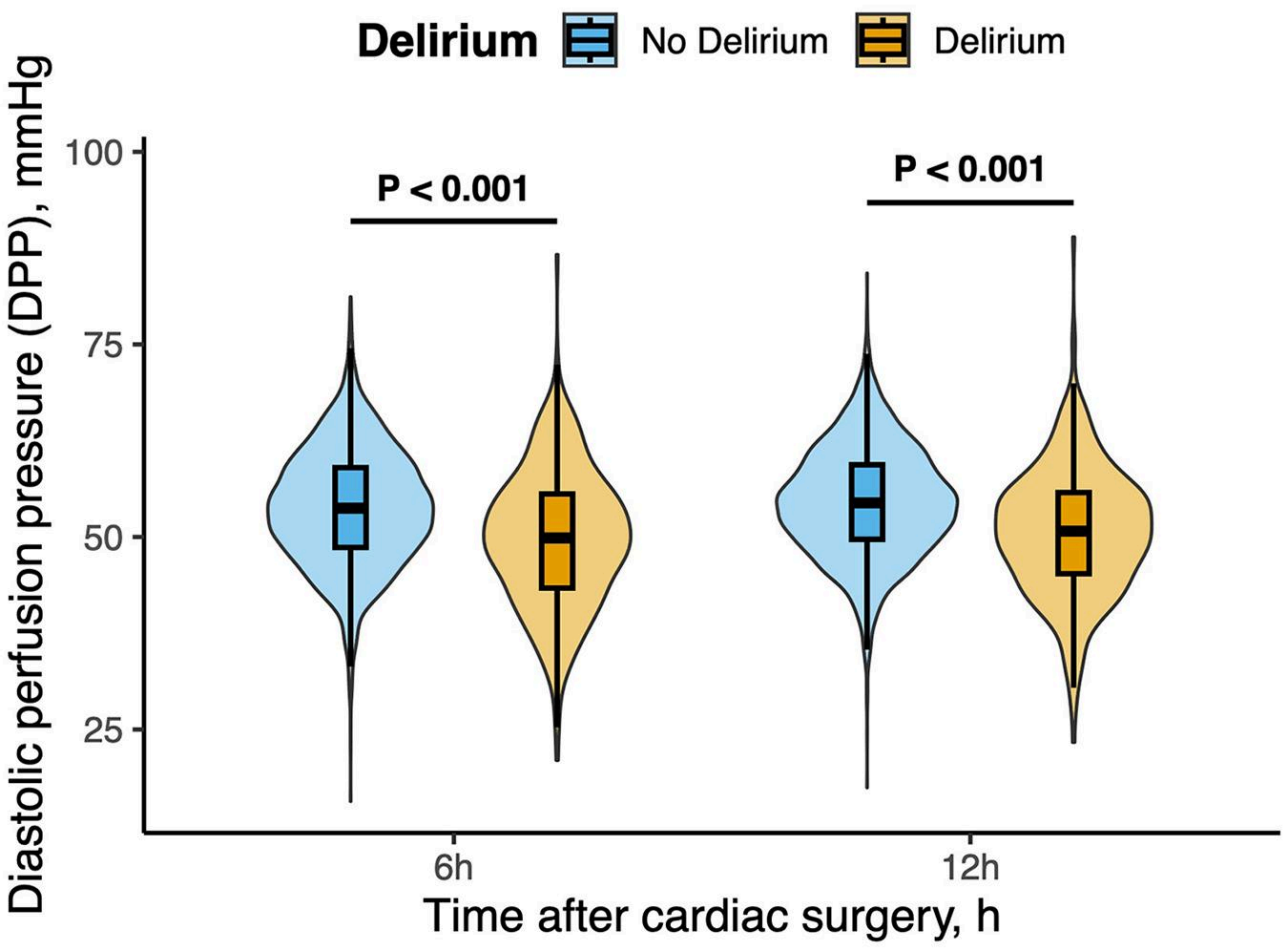
Comparison of diastolic perfusion pressure (DPP) between patients with and without delirium after cardiac surgery. Violin plots show the distribution of DPP values at 6 h and 12 h postoperatively in patients who developed delirium (orange) and those without delirium (blue). The boxes represent the interquartile range (IQR) with the median line shown inside. The width of each violin indicates the kernel density of the data distribution. Patients with delirium had significantly lower DPP at both 6 and 12 h after surgery compared with those without delirium (*P* < 0.001 for both time points).

Table 2 represents the perioperative hemodynamic parameters in the two groups. Patients who developed POD experienced a longer duration of intraoperative hypotension and lower overall blood pressure levels compared with those without POD. However, the time-weighted average thresholds of blood pressure below 65 mmHg, 60 mmHg, 55 mmHg, and 50 mmHg did not differ significantly between the two groups. Postoperatively, patients with POD exhibited higher HR and CVP, along with lower SAP, DAP, MAP, and DPP values at both 6 and 12 h after surgery. Violin plots illustrate the distribution of DPP at 6 [DPP_6h_mean: 50 [43–56] mmHg vs. 54 [49–50] mmHg, *P* < 0.001] and 12 h [DPP_12h_mean: 51 [45–56] mmHg vs. 54 [50–59] mmHg, *P* < 0.001] postoperatively in patients with and without POD (Figure 2). DPP values were significantly lower in the POD group at both time points.

**Table 2:** Perioperative hemodynamic variables of the patients with or without postoperative delirium after cardiac surgery.

Variable	No Delirium (*n* = 4,066)	Delirium (*n* = 352)	*P* value
Intra-operative hemodynamic indexes			
AUT_65	1,455.0 [950.0–2,075.0]	1,777.5 [1,160.0–2,556.2]	<0.001
AUT_60	925.0 [565.0–1,378.8]	1,132.5 [680.0–1,721.2]	<0.001
AUT_55	550.0 [300.0–885.0]	675.0 [358.8–1,081.2]	<0.001
AUT_50	295.0 [130.0–540.0]	360.0 [155.0–651.2]	0.004
BP_65_time, min	115.0 [85.0–155.0]	140.0 [100.0–190.0]	<0.001
BP_60_time, min	80.0 [55.0–115.0]	105.0 [70.0–145.0]	<0.001
BP_55_time, min	55.0 [35.0–85.0]	70.0 [45.0–101.2]	<0.001
BP_50_time, min	35.0 [20.0–55.0]	45.0 [20.0–70.0]	<0.001
TWA_BP_65	12.3 [10.1–15.0]	12.4 [10.1–15.1]	0.868
TWA_BP_60	10.9 [8.6–13.6]	10.7 [8.5–13.4]	0.364
TWA_BP_55	9.3 [7.0–12.3]	9.2 [7.1–12.2]	0.619
TWA_BP_50	7.8 [5.3–11.4]	8.1 [5.7–10.9]	0.757
BP_mean, mmHg	64.9 [60.8–69.0]	63.8 [59.4–67.4]	<0.001
Postoperative hemodynamic indexes			
HR_6h_mean, bpm	84 [78–90]	86 [80–95]	<0.001
SAP_6h_mean, mmHg	113 [106–119]	111 [104–118]	0.005
DAP_6h_mean, mmHg	61 [56–66]	58 [53–64]	<0.001
MAP_6h_mean, mmHg	78 [74–83]	76 [71–81]	<0.001
CVP_6h_mean, mmHg	7 [6–9]	8 [6–10]	<0.001
DPP_6h_mean, mmHg	54 [49–59]	50 [43–56]	<0.001
SpO_2__6h_mean, %	100 [99–100]	100 [99–100]	0.022
HR_12h_mean, bpm	86 [80–91]	86 [80–95]	0.011
SAP_12h_mean, mmHg	115 [108–122]	113 [107–121]	0.023
DAP_12h_mean, mmHg	62 [57–66]	58 [54–64]	<0.001
MAP_12h_mean, mmHg	80 [75–84]	77 [72–82]	<0.001
CVP_12h_mean, mmHg	7 [6–9]	8 [6–10]	<0.001
DPP_12h_mean, mmHg	54 [50–59]	51 [45–56]	<0.001
SpO_2__12h_mean, %	100 [99–100]	100 [99–100]	0.860

AUT_65, the area under blood pressure (BP) 65 mmHg-time curve; AUT_60, the area under BP 60 mmHg-time curve; AUT_55, the area under BP 55 mmHg-time curve; AUT_50, the area under BP 50 mmHg-time curve; BP_65_time, time of BP <65 mmHg; BP_60_time, time of BP <60 mmHg; BP_55_time, time of BP <55 mmHg; BP_50_time, time of BP <50 mmHg; TWA_BP_65, time-weighted average threshold value for BP <65 mmHg; TWA_BP_60, time-weighted average threshold value for BP <60 mmHg; TWA_BP_55, time-weighted average threshold value for BP <55 mmHg; TWA_BP_50, time-weighted average threshold value for BP <50 mmHg; HR, heart rate; SAP, systolic arterial pressure; DAP, diastolic arterial pressure; MAP, mean arterial pressure; CVP, central venous pressure; DPP, diastolic perfusion pressure; SpO_2_, Peripheral capillary oxygen saturation.

To identify independent risk factors for POD, univariate and multivariate logistic regression analyses were performed, incorporating baseline characteristics and perioperative hemodynamic variables (Table 3). In multivariate analysis, male sex [odds ratio [OR] = 1.93, 95% confidence interval [CI]: 1.36–2.74, *P* < 0.001], age (OR = 1.03, 95% CI: 1.02–1.05, *P* < 0.001), APACHE II score (OR = 1.05, 95% CI: 1.02–1.09, *P* = 0.004), EuroSCORE (OR = 1.06, 95% CI: 1.00–1.12, *P* = 0.037), operation time (OR = 1.01, 95% CI: 1.00–1.02, *P* < 0.001), and DPP_12h_mean (OR = 0.83, 95% CI: 0.73–0.94, *P* = 0.004) were identified as independent predictors of POD. In addition, to enhance clinical interpretability, the effect of DPP was also expressed per 5 mmHg decrease in a separate model.

**Table 3:** Univariate and multivariate logistic regression for predicting postoperative delirium in patients receiving cardiac surgery.

Variable	Univariate		Multivariate	
	OR (95% CI)	*P* value	OR (95% CI)	*P* value
Male	1.82 (1.44–2.34)	<0.001	1.93 (1.50–2.52)	<0.001
Age	1.05 (1.04–1.06)	<0.001	1.03 (1.02–1.05)	<0.001
BMI	1.00 (0.95–1.05)	0.923	1.01 (0.96–1.07)	0.632
APACHE II score	1.15 (1.12–1.19)	<0.001	1.05 (1.02–1.09)	0.004
EuroSCORE	1.21 (1.16–1.27)	<0.001	1.06 (1.00–1.12)	0.037
Operation time	1.01 (1.00–1.01)	<0.001	1.01 (1.00–1.01)	<0.001
CPB time	1.01 (1.01–1.01)	<0.001	1.00 (0.99–1.00)	0.622
Aortic cross-clamp time	1.01 (1.00–1.01)	<0.001	1.00 (0.99–1.01)	0.990
AUT_65	1.00 (1.00–1.00)	<0.001	1.00 (0.99–1.00)	0.304
AUT_60	1.00 (1.00–1.00)	<0.001	1.00 (0.99–1.01)	0.457
AUT_55	1.00 (1.00–1.00)	<0.001	1.00 (0.99–1.01)	0.851
AUT_50	1.00 (1.00–1.00)	0.008	1.00 (0.99–1.00)	0.725
BP_65_time	1.01 (1.00–1.01)	<0.001	1.00 (0.98–1.02)	0.787
BP_60_time	1.01 (1.00–1.01)	<0.001	1.01 (0.98–1.04)	0.508
BP_55_time	1.01 (1.00–1.01)	<0.001	0.99 (0.97–1.02)	0.715
BP_50_time	1.01 (1.00–1.01)	<0.001	1.00 (0.98–1.02)	0.776
TWA_BP_65	1.00 (0.97–1.02)	0.937	1.03 (0.91–1.17)	0.672
TWA_BP_60	0.99 (0.96–1.01)	0.326	0.95 (0.83–1.08)	0.461
TWA_BP_55	0.99 (0.97–1.01)	0.402	0.98 (0.90–1.06)	0.682
TWA_BP_50	1.00 (0.98–1.01)	0.637	1.02 (0.98–1.06)	0.224
BP_mean	0.97 (0.96–0.99)	<0.001	1.00 (0.95–1.04)	0.864
HR_6h_mean	1.02 (1.01–1.03)	<0.001	1.02 (0.99–1.05)	0.129
SAP_6h_mean	0.98 (0.97–0.99)	0.001	1.02 (0.88–1.18)	0.752
DAP_6h_mean	0.94 (0.93–0.96)	<0.001	0.90 (0.65–1.23)	0.531
MAP_6h_mean	0.95 (0.93–0.96)	<0.001	0.97 (0.64–1.52)	0.902
CVP_6h_mean	1.12 (1.08–1.16)	<0.001		
DPP_6h_mean	0.94 (0.92–0.95)	<0.001	1.09 (0.97–1.22)	0.153
HR_12h_mean	1.02 (1.01–1.03)	<0.001	0.99 (0.96–1.02)	0.570
SAP_12h_mean	0.99 (0.97–1.00)	0.016	1.03 (0.86–1.25)	0.757
DAP_12h_mean	0.94 (0.93–0.96)	<0.001	1.34 (0.92–2.00)	0.156
MAP_12h_mean	0.95 (0.93–0.96)	<0.001	0.86 (0.48–1.45)	0.604
CVP_12h_mean	1.15 (1.11–1.12)	<0.001		
DPP_12h_mean	0.93 (0.92–0.95)	<0.001	0.83 (0.73–0.94)	0.004

OR, odds ratio; CI, confidence interval; BMI, body mass index; APACHE II, Acute Physiology, Age, Chronic Health Evaluation II; EuroSCORE, European system for cardiac operative risk evaluation; CPB, cardiopulmonary bypass; AUT_65, the area under blood pressure (BP) 65 mmHg-time curve; AUT_60, the area under BP 60 mmHg-time curve; AUT_55, the area under BP 55 mmHg-time curve; AUT_50, the area under BP 50 mmHg-time curve; BP_65_time, time of BP <65 mmHg; BP_60_time, time of BP <60 mmHg; BP_55_time, time of BP< 55 mmHg; BP_50_time, time of BP <50 mmHg; TWA_BP_65, time-weighted average threshold value for BP <65 mmHg; TWA_BP_60, time-weighted average threshold value for BP <60 mmHg; TWA_BP_55, time-weighted average threshold value for BP <55 mmHg; TWA_BP_50, time-weighted average threshold value for BP <50 mmHg; HR, heart rate; SAP, systolic arterial pressure; DAP, diastolic arterial pressure; MAP, mean arterial pressure; CVP, central venous pressure; DPP, diastolic perfusion pressure.

**Figure 3:**
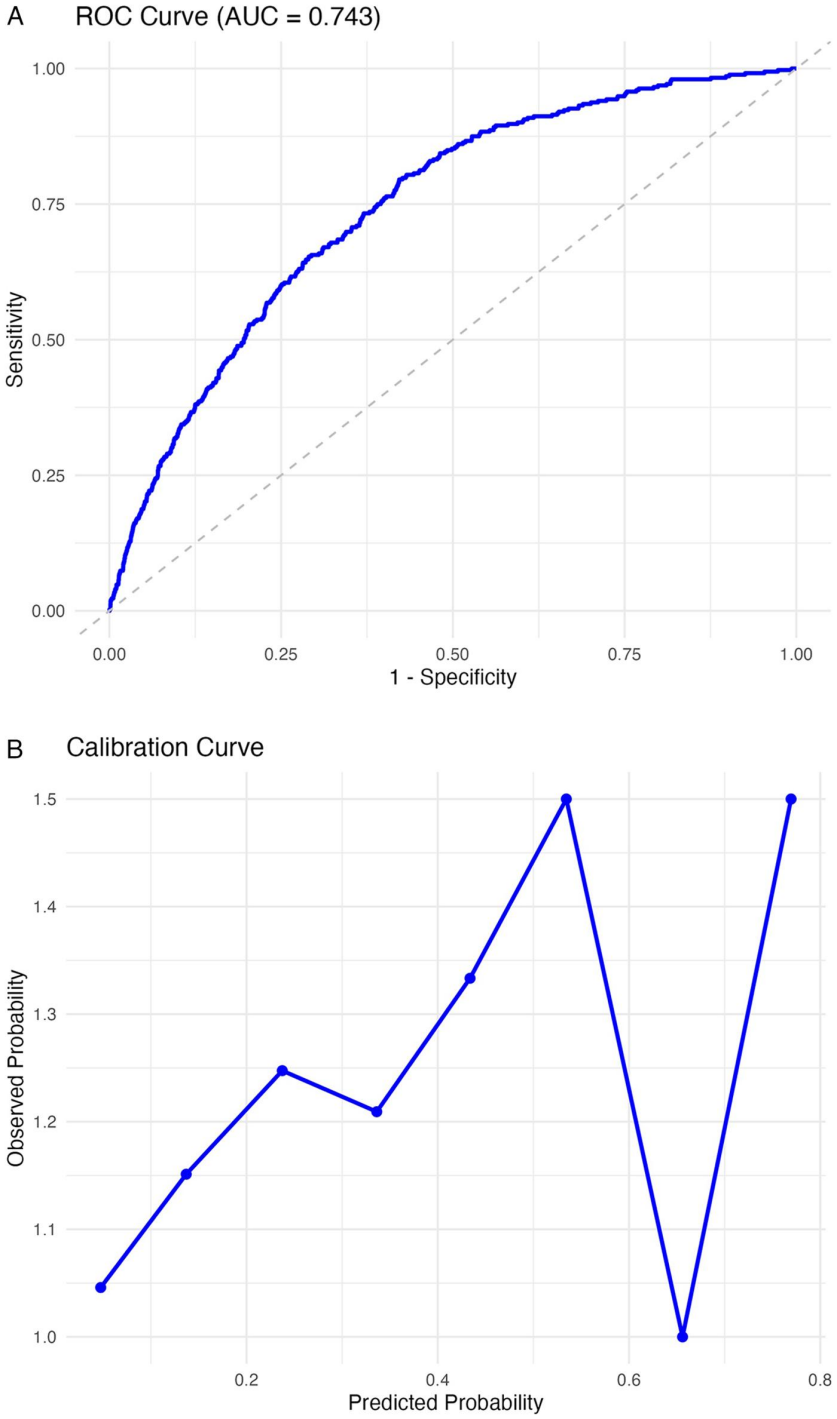
Clinical prediction model for predicting postoperative delirium after cardiac surgery. **(A)** Receiver operating characteristic (ROC) curve of the clinical model for predicting postoperative delirium. The model demonstrated good discriminative ability with a high area under the curve (AUC). **(B)** Calibration curve of the clinical model for predicting postoperative delirium. The predicted probabilities were consistent with the observed outcomes, indicating good model calibration. **(C)** Nomogram of the multivariable logistic regression model for predicting postoperative delirium. Bar plot showing the relative importance of predictor variables in the multivariable logistic regression model for postoperative delirium. The variable male had the greatest contribution to the model, followed by APACHE II score, age, operation time, and DPP_12h_mean. **(D)** Decision curve analysis (DCA) of the clinical model for predicting postoperative delirium. The red line represents the clinical model, while the gray and black lines indicate the “All” and “None” strategies, respectively. The clinical model demonstrates a higher net benefit across a wide range of threshold probabilities, supporting its clinical usefulness.

A clinical prediction model was subsequently constructed incorporating male sex, age, APACHE II score, operation time, and DPP_12h_mean. The receiver operating characteristic (ROC) curve demonstrated good discrimination with an area under the ROC curve (AUROC) of 0.743 (*P* < 0.001) (Figure 3A). The model demonstrated moderate explanatory power, with a Nagelkerke R² of 0.12. This level of explanatory power is consistent with previous clinical prediction models for POD. The calibration curve indicated good agreement between predicted and observed probabilities, with a slight overestimation in the low-risk range but adequate calibration across medium-to-high risk levels (Figure 3B).

**Figure:**
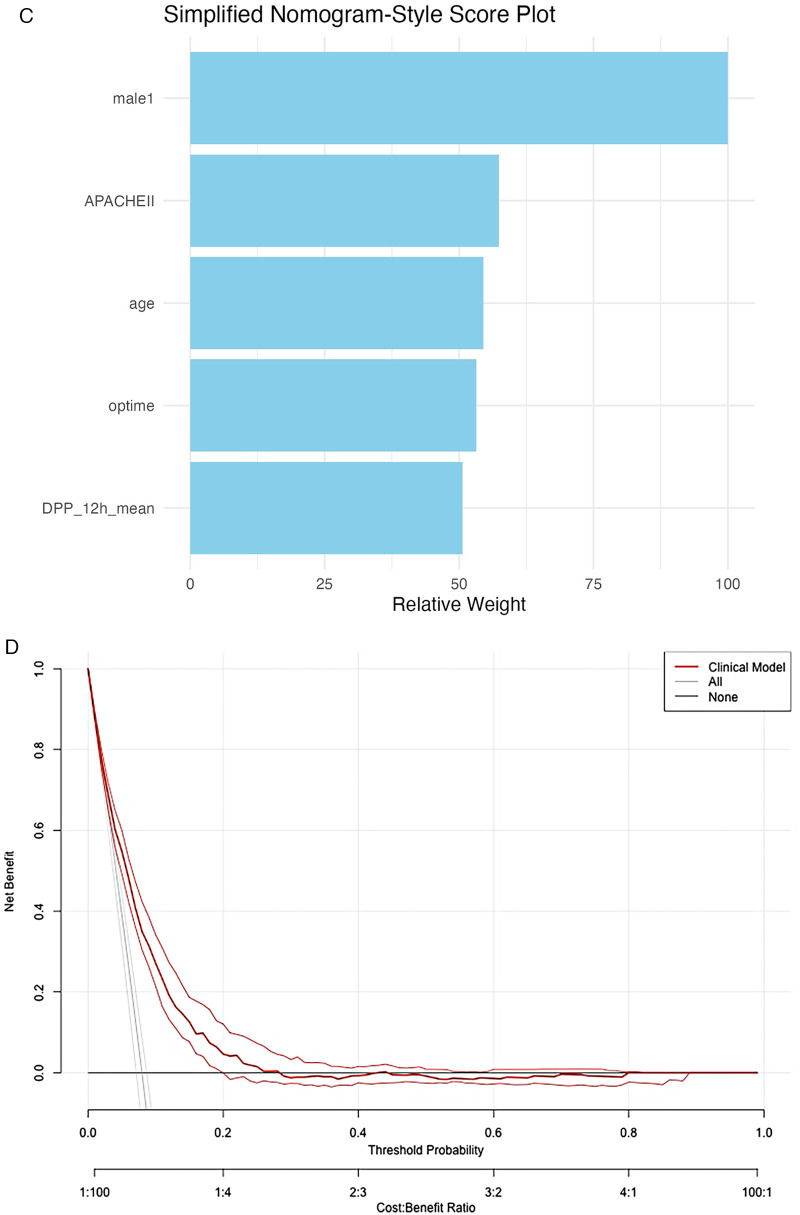


A nomogram derived from the multivariable logistic model illustrated the relative contribution of each predictor variable (Figure 3C). Among these, male sex contributed most substantially to the risk score, followed by APACHE II score, age, operation time, and DPP_12h_mean. Decision curve analysis (Figure 3D) demonstrated that the clinical prediction model provided greater net benefit than either treat-all or treat-none strategies across a broad range of threshold probabilities, confirming its clinical utility for POD prediction.

### Association between DPP and POD

**Figure 4:**
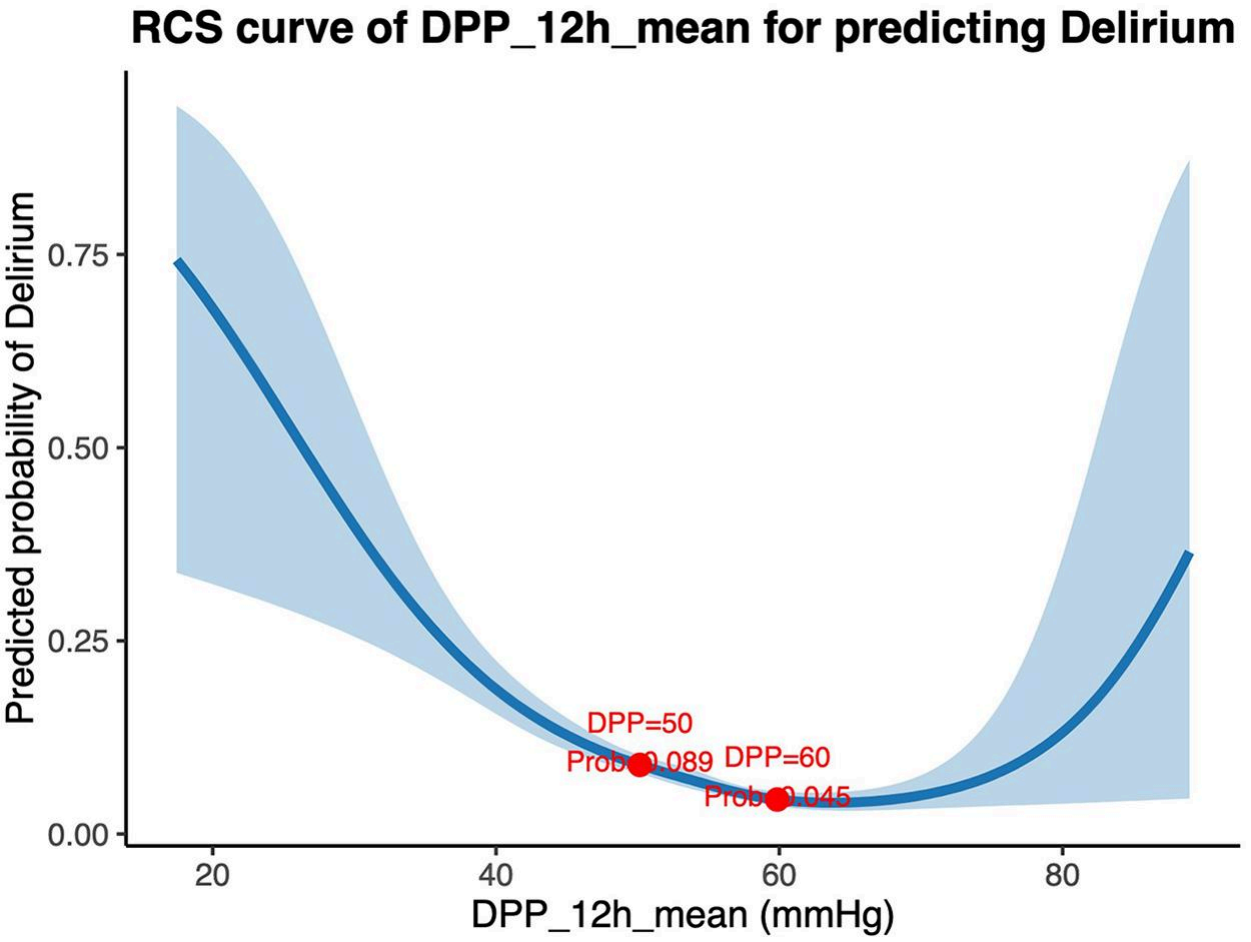
Restricted cubic spline (RCS) curve of DPP_12h_mean for predicting postoperative delirium. The RCS curve shows the nonlinear relationship between the mean diastolic perfusion pressure over 12 h (DPP_12h_mean) and the predicted probability of postoperative delirium. The solid blue line represents the estimated odds of delirium, and the shaded area indicates the 95% confidence interval. The red dots mark specific DPP_12h_mean values (50 mmHg and 60 mmHg) with their corresponding predicted probabilities (*P* = 0.089 and 0.045, respectively).

RCS analysis was used to further explore the relationship between DPP_12h_mean and the risk of POD (Figure 4). A nonlinear association was observed between mean diastolic perfusion pressure over 12 h and the predicted probability of delirium. Results indicated that DPP_12h_mean values of 50 mmHg and 60 mmHg corresponded to predicted probabilities of 0.089 and 0.045, respectively, suggesting that the risk of POD increased markedly when DPP_12h_mean fell below 50 mmHg.

Subsequent logistic regression confirmed that DPP_12h_mean remained an independent predictor of POD after adjusting for age, sex, APACHE II score, and operation time (Table 4). The risk of delirium increased by approximately 20.2% for every 5 mmHg decrease in DPP_12h_mean (OR = 0.80, 95% CI: 0.74–0.86, *P* < 0.001).

**Table 4:** Logistic regression analysis of DPP_12h_mean for predicting postoperative delirium in patients receiving cardiac surgery.

Variable	Univariate		Multivariate		Interpretation
	OR (95%CI)	*P* value	OR (95%CI)	*P* value	
DPP_per5	0.70 (0.65–0.76)	<0.001	0.80 (0.74–0.86)	<0.001	Per 5 mmHg decrease in DPP, risk of Delirium increases by 20.2%.
Age	1.05 (1.04–1.06)	<0.001			
Male	1.83 (1.44–2.34)	<0.001			
Operation time	1.01 (1.01–1.01)	<0.001			
APACHE II score	1.15 (1.12–1.19)	<0.001			

DPP, diastolic perfusion pressure; OR, odds ratio; CI, confidence interval.

### Subgroup analysis

**Figure 5:**
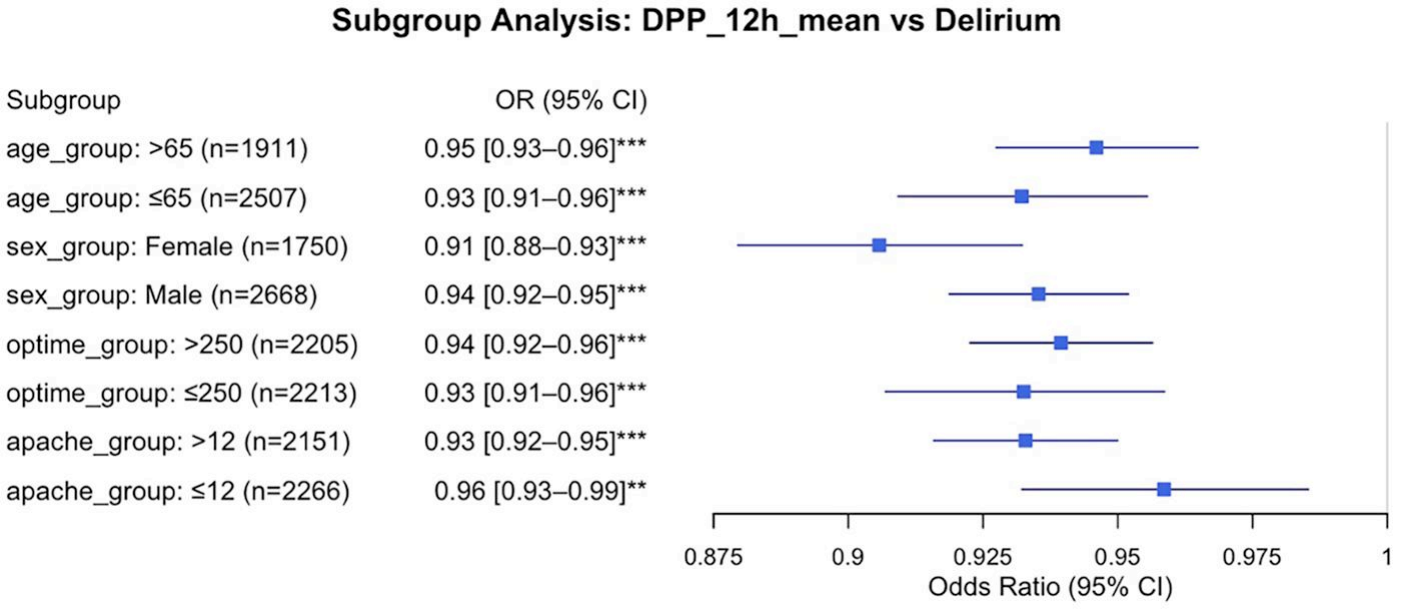
Subgroup analysis of DPP_12h_mean and postoperative delirium. Forest plot showing the association between mean diastolic perfusion pressure over 12 h (DPP_12h_mean) and postoperative delirium across predefined subgroups. Odds ratios (ORs) and 95% confidence intervals (CIs) were estimated using logistic regression. The inverse association between DPP_12h_mean and delirium risk remained consistent across all subgroups.

Subgroup analyses were conducted to assess the robustness of the association between DPP_12h_mean and POD across different clinical strata (Figure 5). The inverse relationship between DPP_12h_mean and the risk of POD remained consistent across age, sex, operation time, and APACHE II score subgroups, indicating the stability and generalizability of the observed association.

## Discussion

In this large retrospective cohort study, we demonstrated that lower DPP within the first 12 h after cardiac surgery was independently associated with an increased risk of POD. Specifically, each 5 mmHg decrease in DPP was associated with an approximately 20% increase in delirium risk. This association remained consistent across multiple clinically relevant subgroups, supporting the robustness of our findings. Although the explanatory power of the model was moderate (Nagelkerke R² = 0.12), this is expected given the multifactorial nature of POD.

### Comparison with existing literature

Postoperative delirium is increasingly recognized as a multifactorial complication influenced by perioperative physiological disturbances. Recent studies have confirmed that delirium results from a complex interaction of patient vulnerability, surgical stress, and perioperative hemodynamic instability (18). Previous investigations have demonstrated that intraoperative and postoperative hypotension, as well as blood pressure variability, are associated with an increased risk of delirium after cardiac surgery (8–10).

However, these studies have largely focused on MAP, which may not adequately reflect effective organ perfusion, particularly in the presence of elevated venous pressure. Compared with previous studies focusing on MAP or blood pressure variability, our study specifically highlights DPP as a more physiologically relevant parameter reflecting effective organ perfusion. Unlike prior reports that primarily evaluated intraoperative hemodynamics, we focused on the early postoperative period, which may better capture the critical window for delirium development.

Our findings extend the current literature by demonstrating that DPP, defined as the difference between diastolic arterial pressure and CVP, may better reflect the effective perfusion gradient at the organ level. This is consistent with emerging evidence suggesting that diastolic perfusion parameters are closely associated with organ dysfunction and adverse outcomes, including acute kidney injury and mortality (11–13, 16).

In addition, increasing attention has been paid to the role of venous congestion in organ dysfunction. Studies have shown that elevated CVP and impaired venous outflow contribute to reduced effective perfusion and may be associated with neurological complications (19, 20). Furthermore, Benkreira et al. demonstrated that portal vein pulsatility, a marker of systemic venous congestion, is associated with delirium after cardiac surgery (21). These findings support the concept that both arterial inflow and venous outflow are critical determinants of organ perfusion.

### Potential mechanisms

Several mechanisms may explain the association between low DPP and POD. First, reduced DPP may lead to impaired cerebral perfusion, particularly in patients with compromised autoregulation following cardiopulmonary bypass. Recent evidence suggests that cerebral hypoperfusion plays a central role in the pathogenesis of postoperative delirium, especially under conditions of hemodynamic instability (22).

Second, low DPP may reflect a combination of reduced arterial pressure and elevated venous pressure, resulting in cerebral venous congestion. Increased venous pressure may impair cerebral venous return, increase intracranial pressure, and contribute to brain edema, thereby promoting delirium (19, 20).

Third, inadequate perfusion may aggravate systemic and neuroinflammation, which is a key mechanism in delirium development (4, 5). A recent prospective study further demonstrated that neuroinflammatory and neuronal injury biomarkers, including interleukin-6 and phosphorylated tau, are independently associated with postoperative delirium (23). Finally, impaired perfusion and inflammation may disrupt neurotransmitter balance and neuronal function, further contributing to the development of delirium.

### Clinical implications

Our findings have important implications for perioperative hemodynamic management. Unlike traditional parameters such as MAP or CVP alone, DPP integrates both arterial perfusion and venous congestion, providing a more physiologically relevant indicator of effective organ perfusion.

From a clinical perspective, monitoring DPP may improve early risk stratification for POD. In particular, DPP may be useful in guiding individualized hemodynamic management by simultaneously considering arterial pressure optimization and avoidance of venous congestion. Strategies aimed at maintaining adequate DPP—such as optimizing diastolic pressure and minimizing venous congestion—may represent potential approaches to reduce delirium risk. Importantly, DPP is readily available from routine monitoring, making it feasible for clinical application.

The observed nonlinear relationship, with a marked increase in delirium risk below approximately 50 mmHg, further suggests that DPP may serve as a clinically meaningful threshold parameter, although this requires validation in prospective studies.

### Strengths and limitations

This study has several strengths. It includes a large sample size, enhancing statistical power and reliability. Multiple analytical approaches, including multivariable regression and restricted cubic spline analysis, were applied to ensure robustness. In addition, DPP is a simple and clinically accessible parameter, increasing the translational value of our findings.

Several limitations should be acknowledged. First, this was a retrospective single-center study, which may limit generalizability and introduce potential selection bias. Second, although multiple confounders were adjusted for, residual confounding cannot be excluded. Third, although anesthesia management was relatively standardized, detailed data on anesthetic depth and drug dosing were not available, which may influence both hemodynamic stability and delirium risk. In addition, ASA physical status classification was not available in the database and therefore could not be included in the analysis, which may have introduced residual confounding. Fourth, DPP was used as a surrogate for organ perfusion, and direct measurements of cerebral perfusion were not available. Fifth, DPP was assessed only during the early postoperative period. Finally, due to the observational design, causal relationships cannot be established.

### What this study adds

This study provides novel evidence that early postoperative DPP is independently associated with delirium after cardiac surgery. By integrating both arterial pressure and venous congestion, DPP may represent a more physiologically meaningful indicator of effective organ perfusion than conventional parameters such as MAP, thereby offering a potential new target for perioperative hemodynamic management.

### Future directions

Future studies should focus on prospective multicenter validation and interventional trials to determine whether targeting DPP can reduce delirium incidence. Integration with cerebral perfusion monitoring and individualized hemodynamic targets represents an important direction for future research.

## Conclusion

Lower postoperative diastolic perfusion pressure is independently associated with an increased risk of postoperative delirium after cardiac surgery. DPP may serve as a clinically useful marker reflecting effective organ perfusion. Optimization of DPP may represent a potential strategy for reducing delirium risk; however, this requires confirmation in prospective interventional studies.

## Data Availability

The raw data supporting the conclusions of this article will be made available by the authors, without undue reservation.

## References

[1] Fang Y Tang X Gao Y Xie H Shen Y Peng M Association between blood urea nitrogen and delirium in critically ill elderly patients without kidney diseases: a retrospective study and mendelian randomization analysis. CNS Neurosci Ther. (2025) 31:e70201. 10.1111/cns.70201 39754021 PMC11702503

[2] Petersson NB Hansen MH Hjelmborg JVB Instenes I Christoffersen AS Larsen KL Incidence and assessment of delirium following open cardiac surgery: a systematic review and meta-analysis. Eur J Cardiovasc Nurs. (2024) 23:825–32. 10.1093/eurjcn/zvae066 38695330

[3] Wang Y Wang B. Risk factors of delirium after cardiac surgery: a systematic review and meta-analysis. J Cardiothorac Surg. (2024) 19:675. 10.1186/s13019-024-03156-1 39707458 PMC11661048

[4] Fan YY Luo RY Wang MT Yuan CY Sun YY Jing JY. Mechanisms underlying delirium in patients with critical illness. Front Aging Neurosci. (2024) 16:1446523. 10.3389/fnagi.2024.1446523 39391586 PMC11464339

[5] Li Z Zhu Y Kang Y Qin S Chai J. Neuroinflammation as the underlying mechanism of postoperative cognitive dysfunction and therapeutic strategies. Front Cell Neurosci. (2022) 16:843069. 10.3389/fncel.2022.843069 35418837 PMC8995749

[6] Ogawa M Izawa KP Satomi-Kobayashi S Tsuboi Y Komaki K Gotake Y Impact of delirium on postoperative frailty and long term cardiovascular events after cardiac surgery. PLoS One. (2017) 12:e0190359. 10.1371/journal.pone.0190359 29287124 PMC5747483

[7] Yokoyama C Yoshitnai K Ogata S Fukushima S Matsuda H. Effect of postoperative delirium after cardiovascular surgery on 5-year mortality. JA Clin Rep. (2023) 9:66. 10.1186/s40981-023-00658-0 37831211 PMC10575819

[8] Othman SMA Aziz MAA Al-Mushiki GMA Sriwayyapram C Okubai T Al-Muwaffaq G Association of postoperative delirium with hypotension in critically ill patients after cardiac surgery: a prospective observational study. J Cardiothorac Surg. (2024) 19:476. 10.1186/s13019-024-02958-7 39090732 PMC11293154

[9] Mohr NL Krannich A Jung H Hulde N von Dossow V. Intraoperative blood pressure management and its effects on postoperative delirium after cardiac surgery: a single-center retrospective cohort study. J Cardiothorac Vasc Anesth. (2024) 38:1127–34. 10.1053/j.jvca.2024.01.027 38369449

[10] Shen X Tao H Chen W Sun J Jin R Zhang W Perioperative blood pressure variability as a risk factor for postoperative delirium in the patients receiving cardiac surgery. BMC Anesthesiol. (2024) 24:424. 10.1186/s12871-024-02817-x 39581994 PMC11587544

[11] Saito S Uchino S Takinami M Uezono S Bellomo R. Postoperative blood pressure deficit and acute kidney injury progression in vasopressor-dependent cardiovascular surgery patients. Crit Care. (2016) 20:74. 10.1186/s13054-016-1253-1 27013056 PMC4806486

[12] Saez de la Fuente I Saez de la Fuente J Martin Badia I Chacon Alves S Molina Collado Z Sanchez-Bayton Griffith M Postoperative blood pressure deficit and acute kidney injury after liver transplant. Exp Clin Transplant. (2022) 20:992–9. 10.6002/ect.2022.0272 36524885

[13] Jin J Yu J Chang SC Xu J Xu S Jiang W Postoperative diastolic perfusion pressure is associated with the development of acute kidney injury in patients after cardiac surgery: a retrospective analysis. BMC Nephrol. (2019) 20:458. 10.1186/s12882-019-1632-3 31823733 PMC6902492

[14] Nguyen DN Huyghens L Parra J Schiettecatte J Smitz J Vincent JL. Hypotension and a positive fluid balance are associated with delirium in patients with shock. PLoS One. (2018) 13:e0200495. 10.1371/journal.pone.0200495 30086136 PMC6080753

[15] Siepe M Pfeiffer T Gieringer A Zemann S Benk C Schlensak C Increased systemic perfusion pressure during cardiopulmonary bypass is associated with less early postoperative cognitive dysfunction and delirium. Eur J Cardiothorac Surg. (2011) 40:200–7. 10.1016/j.ejcts.2010.11.024 21168339

[16] Lim HS Vondrakova D Belohlavek J Rokyta R Ostadal P. Diastolic perfusion pressure predicts response to inotropes and vasopressors and benefit from mechanical circulatory support in cardiogenic shock. Circ Heart Fail. (2025) 18:e012847. 10.1161/CIRCHEARTFAILURE.125.012847 40365681

[17] Alaterre C Fazilleau C Cayot-Constantin S Chanques G Kacer S Constantin JM Monitoring delirium in the intensive care unit: diagnostic accuracy of the CAM-ICU tool when performed by certified nursing assistants - A prospective multicenter study. Intensive Crit Care Nurs. (2023) 79:103487. 10.1016/j.iccn.2023.103487 37451087

[18] Mahmoudi H Chalkias A Moradi A Moradian ST Amouzegar SMR Vahedian-Azimi A. Evaluation of postoperative delirium in cardiac surgery patients with the SDACS screening tool: a multicenter-multiphase study. Perioper Med. (2025) 14:37. 10.1186/s13741-025-00518-8 PMC11948923 40148994

[19] Beaubien-Souligny W Cavayas YA Denault A Lamarche Y. First step toward uncovering perioperative congestive encephalopathy. J Thorac Cardiovasc Surg. (2020) 161:149–53. 10.1016/j.jtcvs.2020.02.146 32624312

[20] Huette P Guinot PG Haye G Moussa MD Beyls C Guilbart M Portal vein pulsatility as a dynamic marker of venous congestion following cardiac surgery: an interventional study using positive end-expiratory pressure. J Clin Med. (2021) 10:5810. 10.3390/jcm10245810 34945106 PMC8706622

[21] Benkreira A Beaubien-Souligny W Mailhot T Bouabdallaoui N Robillard P Desjardins G Portal hypertension is associated with congestive encephalopathy and delirium after cardiac surgery. Can J Cardiol. (2019) 35:1134–41. 10.1016/j.cjca.2019.04.006 31395469

[22] Leivaditis V Sepetis A Mulita F Papatriantafyllou A Mitsos S Tomos P Postoperative delirium after cardiac surgery: psychiatric vulnerability, biological mechanisms, and prevention strategies. Med Sci. (2026) 14:176. 10.3390/medsci14020176 PMC13108142 42029600

[23] Hansen N Knopp CM Esselmann H Celano CM Derad C Asendorf T Prediction of postoperative delirium after cardiac surgery by the interplay between preoperative plasma p-tau181 and IL-6 and heart-brain axis related factors: results from the prospective observational study FINDERI. Mol Psychiatry. (2026) 31(5):2509–19. 10.1038/s41380-025-03412-3 41429986 PMC13099388

